# Laser-Based Strategies to Treat Diabetic Macular Edema: History and New Promising Therapies

**DOI:** 10.1155/2014/769213

**Published:** 2014-09-22

**Authors:** Young Gun Park, Eun Yeong Kim, Young Jung Roh

**Affiliations:** Department of Ophthalmology, Yeouido St. Mary's Hospital, College of Medicine, The Catholic University of Korea, No. 62 Yeouido-dong, Yeongdeungpo-gu, Seoul 150-713, Republic of Korea

## Abstract

Diabetic macular edema (DME) is the main cause of visual impairment in diabetic patients. The management of DME is complex and often various treatment approaches are needed. At the present time, despite the enthusiasm for evaluating several new treatments for DME, including the intravitreal pharmacologic therapies (e.g., corticosteroids and anti-VEGF drugs), laser photocoagulation still remains the current standard in DME. The purpose of this review is to update our knowledge on laser photocoagulation for DME and describe the developments in laser systems. And we will also discuss the new laser techniques and review the latest results including benefits of combined therapy. In this paper, we briefly summarize the major laser therapeutics for the treatment of diabetic macular edema and allude to some future promising laser therapies.

## 1. Introduction

In 2011, an estimated 347 million people worldwide were affected by diabetes, and the number is expected to double by 2030. Diabetic macular edema (DME) is a leading cause of visual impairment in such patients [1] and if left untreated >50% of patients lose more than two lines of visual acuity (VA) within 2 years [2]. DME mostly affects working-age adults, imposing significant burdens both on society and on individual patients; these burdens are expected to increase as the prevalence of diabetes rises [3].

The standard therapy for visual impairment caused by DME is focal and/or grid laser photocoagulation. However, this usually simply stabilizes vision. By applying the Early Treatment Diabetic Retinopathy Study (ETDRS) criteria to patients with visual impairment caused by DME, laser therapy reduced the relative risk of loss of 15 VA letters by 50% compared to deferred treatment [4]. The exact mechanisms by which laser photocoagulation effectively treats DME remain unknown. Both laser-induced destruction of oxygen-consuming photoreceptors and oxygen diffusion through the laser scar to the inner retina may relieve internal retinal hypoxia [5, 6].

However, the era of laser therapy is being rapidly replaced by a new pharmacotherapeutic era associated with rapid improvements in VA. Treatments include intravitreal corticosteroids, intravitreal vascular endothelial growth factor (VEGF) inhibitors, and others under current investigation. In some cases where vitreous traction is demonstrated, the treatment of choice is to perform pars plana vitrectomy (PPV). Some such agents have recently been shown to be superior to laser therapy [7, 8]. However, given that several of these newer agents are available, it can be difficult to individualize treatment options, especially when attempting to minimize cost and simplify retreatment cycles (the number of injections). These concerns, together with the absence of long-term effects on VEGF inhibition, mean that laser photocoagulation continues to be the necessary treatment for DME care. Recently, new (and less destructive) laser modalities including subthreshold micropulse diode (SDM) laser treatment and selective retinal therapy (SRT) have been developed. In the present paper, we summarize the various laser therapeutic options for treating DME and discuss promising laser therapies of the future.

## 2. Conventional Laser Photocoagulation

Laser photocoagulation was inarguably the important treatment method for DME prior to the advent of intravitreal anti-VEGF agents [9]. The efficacy of focal laser treatment may in part be due to its ability to occlude leaking microaneurysms, but the exact mechanism by which focal photocoagulation reduces DME is unknown. Histopathological studies have revealed that such treatment triggers changes in the retina and the retinal pigment epithelium (RPE) [10, 11]. Some authors have suggested that, following the reduction in retinal tissue associated with photocoagulation, autoregulation decreases retinal blood flow to the macula. Such reduced fluid flow is attributable to improvements in oxygenation after photocoagulation [12]. Biochemical and physiological studies have suggested that the mechanism of resolving edema may involve biochemical changes within the RPE [13, 14]. The effectiveness of grid treatment alone, that is, without direct focal treatment of microaneurysms, implies that retinal photocoagulation has an indirect effect on macular edema [15, 16].

The ETDRS trial [4] was the first rigorous, multicenter randomized trial to explore the benefits of laser therapy for DME. Laser photocoagulation was prescribed for all lesions located within two disc diameters of the macular center. Treatment of lesions closer than 500 microns to the macula was not initially planned. However, if vision was less than 20/40, and if retinal edema and leakage persisted, treatment of lesions up to 300 microns from the center was recommended. Three years after randomization, patients who received focal photocoagulation to treat clinically significant macular edema (CSME) exhibited a 50% reduction in the risk of moderate visual loss, compared to controls (12–24%). However, over the same time period, only 3% of patients exhibited VA gains of three or more lines. The suggested ETDRS guidelines [4] for treating DME via laser photocoagulation emphasize direct laser application to leaking microaneurysms combined with grid treatment of areas of diffuse macular leakage and nonperfusion in thickened retinas, especially in those with nonproliferative diabetic retinopathy (NPDR). As initial pan-retinal photocoagulation (PRP) may worsen macular edema by increasing inflammation and the extent of central retinal blood flow [17], the ETDRS recommended combining PRP and focal laser photocoagulation to treat general DME in selected cases with severe NPDR and early-stage proliferative diabetic retinopathy (PDR). Although effective, conventional ETDRS macular photocoagulation causes visible laser scars that may enlarge once the treatment is finished [18]. In addition, the thermal effects of photocoagulation can trigger complications, including choroidal neovascularization (CNV) [19], subretinal fibrosis [20], and visual field loss (central and paracentral scotoma) [15]. Such damage caused by visible end-point laser photocoagulation has encouraged many retinal specialists to seek to reduce the duration of laser exposure and to use less visible clinical endpoints than originally proposed by the ETDRS.

Patient outcomes after application of a modified ETDRS laser protocol or mild macular grid laser (MMG) photocoagulation were evaluated in a randomized controlled trial that included 263 eyes with previously untreated DME, in patients who had 12-month follow-up [21]. Reduction in macular thickness was significantly greater in the group treated with the modified ETDRS laser protocol, but no difference was noted in terms of the mean change in best-corrected VA (0 letters in the ETDRS group and −2 letters in the MMG group, *P* = 0.10), suggesting that modified ETDRS focal photocoagulation should continue to be the standard treatment for DME.

Recently, a randomized controlled trial conducted by the Diabetic Retinopathy Clinical Research Network (DRCR.net) protocol B found that focal/grid photocoagulation was more effective and was associated with fewer side effects than intravitreal injection of triamcinolone acetonide in DME patients at both 2 and 3 years of follow-up [22, 23]. The authors suggested that focal/grid laser treatment should remain to be the standard against which other DME treatments are compared. However, some laser-treated patients (10%) in the DRCR.net protocol I study lost 15 letters or more in VA at 2 years of follow-up [24]. Although it is obviously essential to prevent further loss of vision, the need to restore VA via a novel medical or laser therapy has, until recently, been unmet in DME patients.

## 3. Subthreshold Micropulse Diode Laser Therapy (SDM)

The utility of conventional laser photocoagulation to treat DME has become well established in the time since the ETDRS was reported [4]. However, the procedure produces visible burns in the retina, indicating that the temperature of the tissue is raised to a level sufficient to alter its natural transparency. In other words, photocoagulation, which is currently performed using conventional continuous wave (CW) laser systems, damages the neural retina by inducing the spread of thermal energy from the RPE. Compared to CW treatment, lasers that deliver short pulses (“micropulses”) cause less thermal damage in experimental models of retinal photocoagulation. Moreover, the shorter laser exposure times allow effective treatment of the RPE while at the same time inflicting less damage on the neural retina and the choriocapillaries [25]. The outcomes of “invisible” subthreshold micropulse diode laser (SDM) cannot be discerned using ophthalmic imaging methods such as biomicroscopy, fundus fluorescein angiography (FFA), fundus autofluorescence (FAF), or spectral-domain optical coherence tomography (SD-OCT), because SDM-induced retinal damage is absent. Although the mechanism by which SDM effectively treats DME is unknown, the laser may selectively target the RPE and induce changes in the levels of RPE cytokines [26].

SDM system featuring both 810 nm and 577 nm lasers may, in theory, afford a theoretical advantage because the laser burns will selectively affect the deeper layers, sparing the inner neurosensory retina for the most part. In turn, this should reduce scarring and paracentral scotomas that may arise after treatment [27]. In the micropulse mode, laser energy is delivered via a train of short repetitive pulses (each is typically 100–300 ms in duration) within an “envelope,” the width of which is typically 0.1–0.5 s, and the envelope duration is taken to be the exposure duration. The “ON” time is the micropulse duration. The “OFF” time between successive micropulses allows heat to dissipate in tissues and thermally isolates each pulse [28]. Micropulse power settings as low as 10–25% of visible threshold power have been shown to consistently confine photothermal effects to the RPE, thereby sparing the neurosensory retina. The laser power required for optimal SDM treatment can be estimated by comparing the power that causes a visible retinal burn to that which confines histological damage to the RPE over various duty cycles (the frequencies of the micropulse train) [28]. A previous study [29] explored the long-term safety of SDM by evaluating retinal burn risks when FFA and FAF were used to treat 252 eyes (212 with DME; 40 with branched retinal vein occlusion) followed up for as long as 10 years postoperatively. Inadvertent retinal burns were observed in seven eyes (three Asian, three Hispanic, and one Caucasian). All burns occurred in eyes treated using 10% or 15% duty cycles; no retinal damage was found in any eye treated using a 5% duty cycle. Computational tissue temperature models revealed that SDM performed using a 5% duty cycle triggered an adequate thermal rise in RPE cells and was not lethal to other cells (Table 1).

Micropulse laser treatment of DME has been shown to be as effective as conventional argon laser treatment by several authors. Friberg and Karatza [30] reported that almost 70% of patients experienced clinical resolution of DME by 6 months after SDM photocoagulation, and VA either improved or stabilized in 80% of eyes. Luttrull and Musch [31] found that VA was either stable or improved in 85% of eyes at 12.2 months, and DME was reduced in 96% of eyes. Also, no marked adverse effect of the technique has been reported.

Venkatesh et al. [32] conducted a prospective randomized study using multifocal electroretinography (MfERG) to assess the efficacy of SDM-mediated photocoagulation to treat DME. Thirty-three patients (46 eyes) with CSME were randomized to either an SDM (810 nm) laser or the conventional double-frequency Nd:YAG (532 nm) laser. Six months later, it was concluded that both treatments influenced both VA and central macular thickness. However, MfERG data suggested that use of the SDM laser potentially afforded better preservation of retinal tissue and led to better values for various electrophysiological indices. Many commercial micropulse lasers are available at wavelengths of 532 nm, 577 nm, 586 nm, 660 nm, and 810 nm.

In summary, the cited reports show that the SDM laser is as effective as a conventional laser when used to treat DME. Moreover, the attractive safety profile of SDM treatment allows clinicians to offer earlier treatment for DME, thus at a time when such treatment is likely to prevent tissue damage and the development of visual disability.

### 3.1. Selective Retinal Therapy (SRT)

To further reduce adverse effects on the neural retina, it was already suggested in the early 1990s [33, 34] that selective treatment of the RPE should be delivered carefully so as to avoid thermally damaging adjacent photoreceptors or the choroid. Selective retina therapy (SRT) was introduced in the following decade. SRT is thought to cause laser-induced biological stimulation and rejuvenation of the chorioretinal junction [35]. The method differs from SDM in that RPE cells are selectively damaged without affecting the neural retina, the photoreceptors, or the choroid. The goal of SRT is to stimulate RPE cell migration and proliferation into irradiated areas to improve metabolism at diseased sites.

Selective RPE damage is achieved by applying a burst of microsecond laser pulses in the green spectral range; pulse energy is absorbed primarily by melanosomes within RPE cells. If the pulse energy is appropriate, RPE cells are damaged by microvaporization around intracellular melanosomes when the pulse duration is shorter than 5 *μ*s. High peak temperatures that develop around melanosomes during irradiation create short-lived microbubbles that mechanically disrupt RPE cells as the cell volume rises briefly but markedly [36]. Thus, the SRT technique features the use of microsecond-laser pulses to ensure that damage is RPE-selective and to avoid formation of large bubbles associated with a risk of photodisruption of the retina or choroid [37]. The effects of this treatment are ophthalmoscopically invisible, and fluorescein angiography is used to identify damage to the RPE layer after treatment. Intact bystander RPE cells migrate and proliferate to cover laser-induced RPE defects, thereby recreating an intact RPE barrier layer within 7 days.

**Figure 1:**
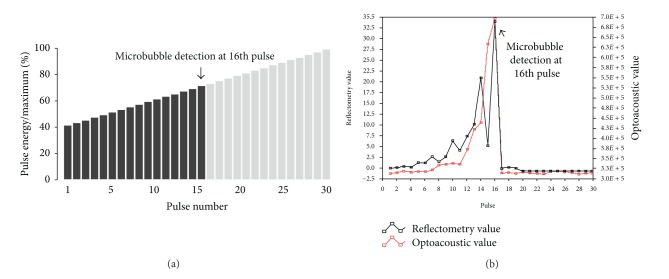
(a) The laser pulse energy was increased stepwise with every pulse by 3% of the dynamic range. In the chosen example, laser irradiation was ceased automatically after the 16th pulse due to detection of microbubble formation. (b) The dual dosimetries show that adequate turnoff system works properly (e.g., turnoff at the 16th pulse).

As transient microbubbles are responsible for the desired effects on RPE cells, it is useful to monitor microbubble development. After each burst, microbubble parameters are evaluated to guard against undertreatment (no microbubbles and thus no effect on the RPE) and overtreatment (large bubbles associated with risks of visible effects and large disruptions). As with SDM, SRT does not cause visible changes in the retina, rendering it difficult to determine when the laser dose is adequate. In efforts to solve this problem, two forms of dosimetry are currently under development. The optoacoustic method features real-time temperature monitoring based on the detection of optically excited thermoelastic pressure waves [35]. Reflectometric methods detect light that is backscattered by the RPE during coagulation. Use of the reflectometric technique with controlled pulse energy ramping is both safe and selective [38] (Figure 1).

The ability of this method to selectively damage RPE cells without injuring photoreceptors has been histologically confirmed at various times after treatment [39]. The first SRT clinical trial using an Nd:YLF laser system and a pulse duration of 1.7 *μ*s (100 pulses, at 100 and 500 Hz) revealed the clinical potential of the technique [40]. Subsequently, the SRT laser parameters were successfully refined. The energy delivered was reduced using even shorter pulses, fewer repetitions, and lower repetition rates [41]. At pulse energies of 450–800 mJ/cm^2^, RPE defects were angiographically demonstrated by detecting fluorescein leakage. However, neither bleeding nor scotoma, as evaluated microperimetrically, was observed, indicating that neither the choroid nor the photoreceptors (resp.) had suffered any adverse effects. During irradiation, the treated locations are ophthalmoscopically invisible, because the effects are both very limited and confined to the RPE.

The precise mechanism of the therapeutic effect is not understood. Several mechanisms have been suggested. It has been histologically shown that the RPE can regenerate following either conventional laser treatment or SRT, reestablishing a normal RPE monolayer [42]. One theory suggests that the beneficial effects of photocoagulation are associated with the establishment of a new RPE cell barrier, with subsequent restoration of the RPE pump and barrier integrity [6].

Such theoretical considerations have led to the development of SRT laser treatments that selectively affect the RPE. Briefly, both thermal modeling and studies* in vitro* and* in vivo* have shown that the spatial extent of elevated temperature is reduced when multiple laser pulses of short duration are delivered using a low repetition rate. By employing such parameters, the effects of laser exposure are confined to the principal light-absorbing structures such as the intracellular melanosomes of the RPE; the photoreceptor layer, Bruch's membrane, and the choroid are spared [36].

Several pilot clinical studies have demonstrated the efficacy of SRT used to treat DME, central serous chorioretinopathy, and persistent subfoveal fluid accumulation after rhegmatogenous retinal detachment [43–46]. Roider et al. [43] found that SRT was potentially effective and safe when used to treat clinically significant DME; functional and anatomical improvements or stabilization was noted in 84% of patients. Mean BCVA improved from 43.7 letters at baseline to 46.1 letters at the 6-month follow-up (*P* = 0.02), and improvement of >5 letters, or no deterioration, was noted in 84% of eyes. No adverse effects or pain was recorded during or after treatment.

**Figure 2:**
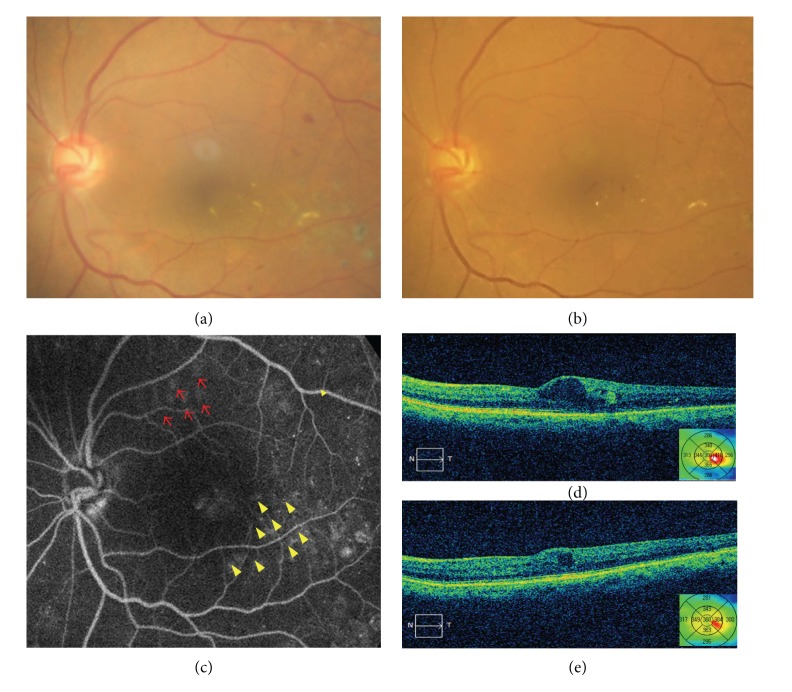
Color fundus photographs showing the reduction in hard exudates before (a) and 3 months (b) after SRT for DME. Laser test spots were applied (red arrow) and SRT treatment was performed (yellow arrowhead). OCT scans showing the reduction of retinal thickness. OCT scan and retinal thickness map before (d) and 3 months after SRT (e).

Although SRT has not yet been commercialized, both optoacoustic systems and reflectometry will help define the energy required for selective RPE damage. Therefore, using a combination of both dosimetric methods to ensure safety, SRT could be an important subthreshold laser treatment modality for DME in the near future (Figure 2).

## 4. Laser Therapy Combined with Pharmacological Treatment

DME is a chronic disease with variable response and clinical manifestations and it does not appear reasonable that a single treatment may be enough for the entire course of the disease. Above all things, laser photocoagulation, the current gold standard of care in DME patients, usually only stabilizes vision. However, as VA improvements after laser therapy occur only very slowly, the addition of laser treatment to the use of pharmacological agents confers an additional benefit in terms of both VA and patient quality-of-life. The available treatments should also make the most of the beneficial effects of each existing approach, exploiting the opportunity for more successful combined therapy. In particular, it is well known that laser treatment can reduce oxygen consumption and influence the RPE in a complex manner.

The introduction of intravitreal anti-VEGF, corticoids (triamcinolone), and steroid implants in DME treatment altered the current treatment protocols. Several studies have compared the effectiveness of new drugs alone with that of laser therapy alone or combined with drugs.

## 5. Intravitreal Anti-VEGF Treatment Alone or Combined with Laser Therapy

### 5.1. Ranibizumab (Lucentis, Genetech, San Francisco, CA)

Ranibizumab is a recombinant humanized monoclonal antibody fragment specific for all isoforms of human VEGF-A and has been approved by the Food and Drug Administration (FDA) for intravitreal injection for the treatment of retinal diseases. Ranibizumab has been evaluated as an adjunct to laser photocoagulation in well-conducted prospective studies such as READ-2 [47] and the DRCRnet protocol I [48], as well as the RESTORE [49].

The READ-2 study [47] showed that patients who received ranibizumab alone (group 1) gained an average of 7.4 letters at 6 months as compared to a 0.5-letter loss in patients receiving macular laser therapy only (group 2) and a 3.8-letter gain in patients receiving both laser treatment and ranibizumab (group 3). At 24 months, and after starting groups 2 and 3 on ranibizumab at 6 months, the mean improvements in the best-corrected visual acuity (BCVA) were 7.7, 5.1, and 6.8 letters in groups 1 to 3, respectively. The optical coherence tomography (OCT) findings, however, did not parallel the visual outcome. The mean foveal thicknesses at 24 months were 340, 286, and 258 *μ*m for groups 1 to 3, respectively. The DRCRnet protocol I trial [48] outcomes indicated that four monthly injections of ranibizumab and then as needed combined with prompt or deferred laser was more effective than prompt laser alone in patients with visual impairment associated with DME (BCVA letter score +9 for the ranibizumab-plus-laser group versus +3 for the laser-alone group; *P* < 0.001).

Recently, DME management has shifted progressively to feature intravitreal drug therapy, usually delivered via injection every 4–6 weeks. To limit the treatment burden associated with frequent injections, several studies have explored combination regimens featuring macular laser photocoagulation and anti-VEGF drug delivery to determine if the number of interventions could be reduced.

Phase III Trials in the Ranibizumab Monotherapy or Combined with Laser versus Laser Monotherapy for Diabetic Macular Edema (RESTORE) study [49]; three monthly injections of ranibizumab 0.5 mg and then as needed either alone or combined with laser therapy was more effective than laser alone in patients with DME. However, no efficacy differences were detected between the ranibizumab alone and ranibizumab-plus-laser arms of this trial. But a subgroup analysis of data from this trial indicated that patients with retinal thicknesses ≤ 300 *μ*m enjoyed similar outcomes after either laser or anti-VEGF monotherapy, whereas patients with thicker retinas benefited most from anti-VEGF monotherapy.

The results suggest that initial anti-VEGF monotherapy may reduce retinal thickness, thereby improving the substrate for subsequent focal laser application, which is most effective when used to treat relatively thin retinas.

### 5.2. Bevacizumab (Avastin, Genetech, San Francisco, CA)

Bevacizumab was approved by the FDA for the treatment of colorectal cancer. It has been used off label in the treatment of wet AMD and other ocular diseases including DME [50, 51]. A recent prospective randomized controlled clinical trial (the BOLT study) found that bevacizumab has a greater effect than macular laser treatment in patients with center-involving persistent CSME [8]. At 12 months, there was a significant difference in the mean BCVA (*P* = 0.0006). At 2 years, the mean BCVA was also increased in the bevacizumab group compared to the macular laser therapy group (*P* = 0.005).

### 5.3. Aflibercept (VEGF Trap-Eye, Regeneron Pharmaceutical, NY)

Aflibercept is the most recent anti-VEGF approved for clinical use. It is a pan-isoform VEGF-A inhibitor with substantially greater binding affinity to VEGF than either bevacizumab or ranibizumab. The DA VINCI study [52, 53], a phase 2 clinical trial, compared different doses and dosing regimens of aflibercept with laser photocoagulation in patients with DME: aflibercept 0.5 or 2 mg every 4 weeks, 2 mg every 8 weeks, or 2 mg as needed after 3 initial monthly injections or macular laser treatment. At 52 weeks, the mean improvement ranged from 9.7 to 12 letters in the aflibercept groups versus −1.3 for laser group. The mean reduction in central retinal thickness in the aflibercept groups ranged from −165.4 to 227.4 versus −58.4 for the laser group.

## 6. Laser Therapy Combined with IVTA

Many clinical trials have investigated the effects of intravitreal triamcinolone acetonide (IVTA) alone or combined with laser therapy in DME. The 3-year follow-up reports involved only patients treated with intravitreal triamcinolone 1 or 4 mg or laser photocoagulation. The mean change in VA from baseline to 3 years was +5 in the laser group and 0 in each triamcinolone group. The VA outcomes slightly favored the laser group [23]. In addition, Se et al. [54] randomized 86 eyes with diffuse DME to receive either IVTA or IVTA followed by grid laser treatment. They found improvement in the VA and central macular thickness in both groups after 3 weeks. After 6 months, however, these improvements were maintained in the combined group only, suggesting that laser treatment acted synergistically with IVTA, resulting in an increased duration of the effect attributable to IVTA [54].

The 1-year mean change in the VA from baseline was significantly greater in the ranibizumab + prompt laser and ranibizumab + deferred laser groups, but not in the triamcinolone + prompt laser group, compared with the sham + prompt laser group. By contrast, in pseudophakic eyes, the VA improvement in the triamcinolone + prompt laser group appeared comparable to that in the ranibizumab groups [55].

## 7. Laser Therapy Combined with Steroid Implants

The major limitation of using IVT as adjunctive therapy for DME is the short duration of action and the need for multiple injections that carry the risk of cataract and glaucoma [56]. The recent availability of corticosteroid implants has allowed new approaches to treating DME with combined therapy [57]. Several intravitreal steroid-releasing implants have been designed to facilitate long-term drug delivery to the macular region. These include nonbiodegradable and biodegradable implants containing dexamethasone, fluocinolone acetonide, and triamcinolone acetonide. The sustained-release biodegradable dexamethasone intravitreal implant (Ozurdex, Allergan, Irvine, CA) is receiving attention from medical professionals.

In the multicenter Ozurdex assessment for DME (MOZART study) [58], the mean improvement in the BCVA from baseline was 7.6 letters at 6 months. A gain greater than 15 letters was found in 27% of the patients at 6 months. In addition, the average CRT decrease was 135 *μ*m at 6 months. The mean rate of injection was 1.2 at 6 months, with an average of 5.4 months for reinjection. Side effects are rare and manageable. The use of injectable, sustained-release steroid implants might be considered as an optional treatment and combined with laser treatment to achieve a beneficial long-term effect in DME.

Anti-inflammatory drugs, especially corticosteroids, can counter the various inflammatory reactions associated with diabetic retinopathy, and anti-VEGF drugs inhibit the effects of VEGF on retinal and vascular structures. Recently, DME management has shifted progressively towards intravitreal drug therapy, usually delivered via injection every 4–6 weeks. In the case of some sustained delivery implants, the injections can be given at intervals of up to several months.

To limit the treatment burden associated with frequent injections, several studies have explored regimens combining macular laser photocoagulation with anti-VEGF or anti-inflammatory drug delivery to determine if the number of interventions could be reduced [24, 47, 49, 55].

## 8. Discussion

The treatment of DME is evolving rapidly. The era of laser therapy is being quickly replaced by a new era of pharmacotherapy. Several pharmacotherapies have recently been developed to treat retinal vascular diseases including DME. Several types of intravitreal drugs and sustained delivery devices have undergone phase 3 testing and others are currently being evaluated. The results of clinical trials have shown that the therapeutic effects of intravitreal agents such as anti-VEGF and steroid implants are short-term compared to those of laser therapy. Thus, frequent injections are needed to treat diseases that are chronic and recurrent. Subthreshold laser treatment is easy to deliver and is not associated with any of the serious complications of intravitreal injection (endophthalmitis, retinal detachment, and glaucoma).

Several new lasers used to treat DME are attracting increasing attention, as they are yielding promising results. Laser treatment has been shown to be an effective treatment option, at least compared to ETDRS photocoagulation in patients without pericentral scotoma (which reduces retinal function). In terms of expansion of indications, subthreshold lasers (SDM and SRT) may be valuable for treating subclinically significant DME that is diagnosed early, thus prior to symptomatic and irreversible visual loss, using new high-resolution imaging techniques such as SD-OCT. Such techniques may make it possible to perform safe early interventions to reduce disease risk and the rate of disease progression, in turn reducing inflammation and improving the health of the RPE.

The expansion of retinal phototherapeutic techniques may lead to the development of new treatment strategies and make it possible to manage, or even prevent, DME. Lasers may be used alone or in combination with pharmacological therapies. Such treatment options will play important roles in the complex management of DME.

## Figures and Tables

**Table 1:** Comparison of subthreshold micropulse laser (SDM) systems.

Model name (manufacturer)	PASCAL streamline 577 (Topcon)	IQ810 (Iridex)	2RT (Ellex)
Category	End point treatment	Subthreshold micropulse laser	Retinal rejuvenation therapy
Laser type	Optically pumped semiconductor	Diode	Q-switched green
			YAG laser
Wavelength	577 nm	810 nm	532 nm (green)
Pulse duration	10 to 1000 ms	CW pulse: 10–9000 ms	3 ns
		Micropulse: 0.025–1 ms	
Power	30–150 mW	0–2000 mW	Energy: 0.6–1.2 mJ
	150–2000 mW		Fluence: 200 mJ/cm^2^
Spot size	60/100/200/400 *μ*m	125 *μ*m	400 *μ*m
Dosimetry	N/A	N/A	N/A
